# Direct Writing of a Titania Foam in Microgravity for Photocatalytic Applications

**DOI:** 10.1021/acsami.3c09658

**Published:** 2023-09-28

**Authors:** G. Jacob Cordonier, Kyleigh Anderson, Ronan Butts, Ross O’Hara, Renee Garneau, Nathanael Wimer, John M. Kuhlman, Konstantinos A. Sierros

**Affiliations:** † Department of Mechanical and Aerospace Engineering, West Virginia University, Morgantown, West Virginia 26506, United States

**Keywords:** Direct foam writing, additive manufacturing, microgravity, titanium dioxide, photocatalysis

## Abstract

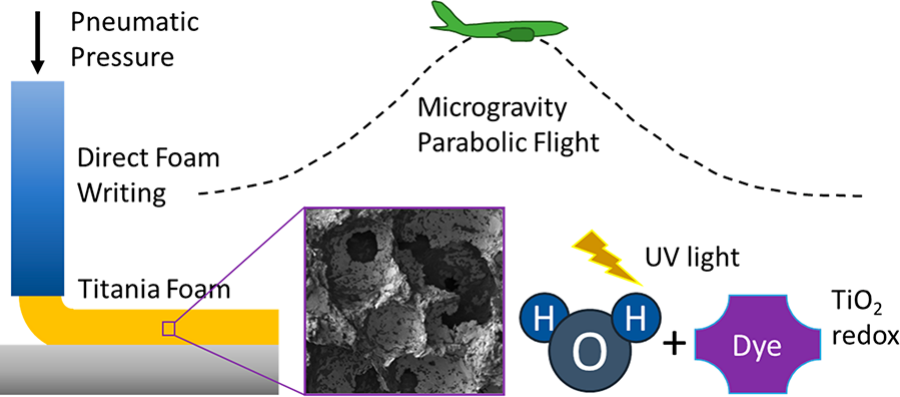

This work explores the potential for additive manufacturing to be used to fabricate ultraviolet light-blocking or photocatalytic materials with in situ resource utilization, using a titania foam as a model system. Direct foam writing was used to deposit titania-based foam lines in microgravity using parabolic flight. The wet foam was based on titania primary particles and a titania precursor (Ti (IV) bis(ammonium lactato) dihydroxide). Lines were also printed in Earth gravity and their resulting properties were compared with regard to average cross-sectional area, height, and width. The cross-sectional height was found to be higher when printing at low speeds in microgravity compared to Earth gravity, but lower when printing at high speeds in microgravity compared to Earth gravity. It was also observed that volumetric flow rate was generally higher when writing in Earth gravity compared to microgravity. Additionally, heterogeneous photocatalytic degradation of methylene blue was studied to characterize the foams for water purification and was found to generally increase as the foam heat treatment temperature increased. Optical and scanning electron microscopies were used to observe foam morphology. X-ray diffraction spectroscopy was used to study the change in crystallinity with respect to temperature. Contact angle of water was found to increase on the surface of the foam as ultraviolet light exposure time increased. Additionally, the foam blocked more ultraviolet light over time when exposed to ultraviolet radiation. Finally, bubble coarsening measurements were taken to observe bubble radius growth over time.

## Introduction

With the rising interest in Lunar and Martian exploration in recent years, there has been a significant increase in attention given to *in situ* resource utilization (ISRU).^1−6^ Although this concept is not new,^7,8^ advances in additive manufacturing capabilities, particularly in space,^9−11^ have expanded the potential for additive manufacturing technologies to play a significant role in long-term space missions. By using materials available and manufacturing methods feasible in these environments, space missions could be performed at a lower cost through reductions in launch mass, as well as for longer periods of time. Avenues for taking advantage of in-space manufacturing include ultraviolet (UV) radiation shielding and water purification. One mitigation method that can be employed is the application of UV radiation shielding material to protect astronauts or sensitive electronic equipment from this harmful radiation. Titanium dioxide (TiO_2_) could be used for both water purification^12−14^ and UV shielding.^15−17^ TiO_2_ is particularly attractive as deposits have been observed in the lunar regolith, making ISRU a possibility.^18−20^ TiO_2_ has also been incorporated into foams which hold an advantage over thin films due to the increased surface area and the porosity of the cell structure, offering more adsorption sites for reactions to occur.^21,22^ Additionally, certain foams have been shown to be more stable in a microgravity environment versus that of Earth gravity due to the lack of gravitational forces causing drainage-induced degradation^23,24^ and in-space manufactured structures using these foams could demonstrate improved performance compared to those manufactured on Earth.

Additive manufacturing in microgravity has been receiving increased consideration as an avenue for in-space fabrication of, for example, surgical instruments^10^ and scaffold-free engineered biomimetic tissues.^25^ Direct Foam Writing (DFW) is a method of additive manufacturing that has been demonstrated in a microgravity environment with a titania foam.^26^ Furthermore, DFW of porous, ceramic materials has been demonstrated to be beneficial in artificial photosynthetic systems,^27^ high-temperature insulation,^28^ high-temperature particulate matter filtration,^29^ lattices with tunable stiffnesses, strengths, and energy absorptions,^30^ and inorganic semiconductor photocatalysis.^31^ However, there is a knowledge gap on the DFW printing behavior of porous materials in a microgravity environment. The intent of this study is to perform a comprehensive investigation using data collected from DFW titania foams during parabolic flight to examine the differences between morphologies of foams written in microgravity and those written in Earth gravity conditions, as well as characterization of various foam properties in Earth conditions.

DFW was employed to deposit lines of a titania particle-based foam in both microgravity and Earth-gravity environments onto glass substrates using a custom-made three-dimensional (3D) printing system (Supporting Information Figure S1) housed in an experimental payload frame and flown onboard a parabolic flight aircraft (Zero-G Corp.). The written lines of foam are characterized using optical profilometry with regards to cross-sectional area, width, height, and roughness. The foam rheology and coarsening behavior are examined. Additionally, the foam is investigated using optical microscopy and scanning electron microscopy (SEM) to study its morphology. For temperature-related structure changes, thermogravimetric analysis (TGA) and X-ray diffraction (XRD) are used. UV-induced hydrophobicity of foam films is examined using contact angle. Heterogeneous photocatalytic degradation of methylene blue has been carried out to characterize the water purification properties of the foam, and UV–vis absorption has been used to determine how light interacts with foam films.

## Materials and Methods

### Titania Foam Ink Synthesis

The foam was prepared by first mixing the aqueous and oil phases separately, then combining them and frothing the resulting emulsion to incorporate air bubbles. For the aqueous phase, Ti(IV) bis(ammonium lactato) dihydroxide (TALH, 50% wt. in H_2_O, Aldrich), deionized (DI) water, and TiO_2_ nanoparticles (Titanium(IV) oxide nanopowder, 21 nm primary particle size, Sigma-Aldrich), were mixed and stirred magnetically in a beaker for 15 min. The mixture was then set in a sonication water bath at room temperature for 15 min before being stirred again for 15 min and then sonicated for a final 15 min. The mixture was then stirred for 1 h to ensure thorough dispersion of the TiO_2_ particles. The weight % for the aqueous phase’s constituent parts is 25.8% TiO_2_, 58.3% DI Water, and 15.9% TALH.

For the oil phase, stearic acid (97%, Acros Organics), polysorbate 60 (P60, Thermo Scientific), and glycerol (>99%, Sigma-Aldrich) were heated in a beaker covered in parafilm at 80 °C. Once melted, the mixture was stirred until it became homogeneous. The weight % for the oil phase’s constituent parts is 30.1% stearic acid, 41.7% P60, and 28.2% glycerol. The aqueous phase was then added to the oil phase dropwise and while stirring slowly to prevent agglomeration of the TiO_2_. The final weight ratio of aqueous to oil phase was 79.7% to 20.3%, respectively. The mixture was then stirred at 350 rpm while continually being heated at 80 °C for 5 min. Finally, the mixture (oil phase + aqueous phase) was frothed with a JJ-1 Accurate Electric Stirrer for 8 min at 1500 rpm to incorporate air bubbles. We note that the foam formulation resembles that of foams fabricated by Torres et al.^22^

### Direct Foam Writing

A custom-made 3D printer was used to deposit the foam patterns (Figure S1). The design and operation of the 3D printer has been described in detail in a previous work.^26^ The foam was extruded through a plastic tapered nozzle (Nordson) with an inner diameter of 580 μm at the tip. The nozzle standoff distance was 508 μm. Nozzle pressure and writing speed were varied between 13.8 and 27.6 kPa and 5–11.31 mm/s, respectively. Printing in microgravity was performed in parabolic flight on a modified Boeing 727–200 operated by Zero Gravity Corporation. The foam lines were printed during 20 s intervals of microgravity flight.

### Scanning Electron Microscopy

A JEOL JSM-7600F scanning electron microscope was used to image cross sections of the foam using secondary electrons. Samples were scanned with a 1.0 kV bias and a working distance of 9.5 mm.

### Viscosity

Apparent viscosity of the foam was measured using a Brookfield LVDV-III+ Programmable Rheometer in ambient conditions with an LV4 spindle in a cup and bob geometry (Brookfield Small Sample Adapter) and a steady shear rate sweep. The approximate sample volume was 3.5 mL.

### TGA Characterization

Thermogravimetric analysis (TGA) of the foam was performed using a Pyris 1 TGA (PerkinElmer) with a heating/cooling rate of 10 °C/min. The foam was held at 600 °C for 30 min before cooling.

### Contact Angle Characterization

Contact angle of deionized (DI) water on doctor bladed films of the titania foam was measured. The foam was doctor bladed onto glass slides at a thickness of 200 μm. One set of samples was heated in a furnace up to 500 °C for 1 h. Another set of samples was exposed to ultraviolet light in a SpectroLINKER XL-1500 Spectroline UV chamber for up to 48 h. The bulbs delivered an average intensity of ∼2800 μW/cm^2^ at 254 nm wavelength. A Thermo Scientific Matrix electronic pipet was used to deposit 2 μL droplets of DI water onto the foam films. A Dino-Lite Edge optical microscope was used to image the droplets. Ten droplets were measured and averaged per film. ImageJ software was used to analyze the images with a low-bond axisymmetric drop shape analysis plugin.^32^

### XRD Characterization

X-ray diffraction (PANalytical X’Pert Pro) was used to quantify the crystalline size of the titanium dioxide particles in both heat-treated and UV-exposed foam film samples doctor-bladed at thicknesses of 200 μm. X’Pert Highscore Plus PANalytical software was used to identify the anatase and rutile phases of the titania.

### UV–visible Spectroscopy Characterization

UV–visible light absorbance of the cured foam was measured using a BioTek Epoch Spectrophotometer. The foam ink was doctor bladed 200 μm thick on a borosilicate slide. The absorbance spectrum of an empty slide was subtracted from the spectrum reading of the sample. The absorbance of the sample was averaged over 10 discrete measurements across the sample. The absorbance spectrum was converted into percent light transmitted using the following equation:
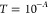
1where *T* is the percent transmittance and *A* is the absorbance.

### Coarsening Image Analysis

Samples of the foam were placed in 4.5 mL cuvettes and imaged with a digital microscope once per hour for a period of 35 days. The images were analyzed in MATLAB to measure the radii of the air bubbles suspended in the ink. First, the images were cropped to the edges of the cuvette of interest. Next, the images were blurred to remove compression artifacts. The blurring used a 2-D Gaussian smoothing kernel with a standard deviation of 2. Then, the images were converted to grayscale. The complements of the images were used so the dark regions of the bubbles were represented with higher pixel values. The contrast was enhanced using a local contrast enhancement algorithm with an edge threshold of 0.5 and an amount of 1. To segment the air bubbles suspended in the ink, the images were binarized using a threshold of 150 (8-bit pixel values, max 255). Next, holes in the binarized image were filled and the region around the border of the cuvette was excluded. The pixel areas of the bubbles in each frame were recorded. For simplicity, it was assumed the bubbles formed perfect circles. The images were scaled using a known distance and the radii were converted from pixel values to millimeters.

### Optical Profilometry

Optical profilometry was performed using a Bruker Contour GT KO Optical Profiler. 3D scans laterally across the printed lines of foam were taken using a green light and a 5× objective. At least 24 cross sections were extracted from each 3D scan to obtain width, height, cross-sectional area, and roughness data using Bruker’s Vision64 software. Selected profiles of the deposited foam lines are shown in Figure S2, illustrating that the foam lines exhibit a roughly semielliptical cross-sectional shape. An unbalanced ANOVA test was performed (Figure S3 and Figure S4) to determine any statistically significant differences in the data.

### Optical Microscopy, Density, and Volumetric Flow Rate

A Keyence VHX-7000 digital microscope was used to capture surface images of doctor-bladed films and three-dimensional (3D) scans of the printed lines of the titania foam. Apparent foam density was measured by printing out lines of foam on glass, weighing the mass of foam printed, and dividing by the volume of the printed line as measured from the 3D scans. An average density of 0.77 ± 0.24 g/mL was found for the titania foam. 3D images were used to measure the volume of the deposited foam lines for volumetric flow rate (*Q*) calculations. Volumetric flow rate was measured experimentally by scanning the volume of a section of a printed line that was printed at a known writing speed and pressure. The volume was then divided by the time taken to deposit the section of the printed line. An example of a Keyence image is depicted in Figure S5.

### Heterogeneous Photocatalytic Degradation of Methylene Blue

Heterogeneous photocatalytic degradation of methylene blue hydrate (Acros Organics) was carried out. Samples of the TiO_2_ foam were doctor bladed onto glass slides in 2.54 mm × 2.54 mm patterns with a thickness of 200 μm and heated at various temperatures up to 600 °C in a furnace for 1 h. The samples were then submerged in beakers containing 20 mL solutions of 10 μM methylene blue in DI water and exposed to UV light in a SpectroLINKER XL-1500 Spectroline UV chamber for up to 150 min. An Evolution 300 UV–vis spectrometer (Thermo Scientific) was used to measure the light absorbance of the solutions in polystyrene cuvettes at 664 nm with a 2 nm bandwidth at 30 min intervals. A baseline measurement of a cuvette filled with DI water was subtracted from each measurement. Each sample was measured five times and averaged. The Beer–Lambert law was assumed to be valid under the testing conditions and the apparent degradation rate constant, *k*_app_, was calculated graphically (see Heterogeneous Photocatalytic Degradation of Methylene Blue in the Results and Discussion section), assuming pseudo-first order kinetics, from the slope of the linear fit of the change in concentration over time using the following equation:
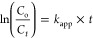
2where *C*_o_ is the initial dye concentration and *C*_*t*_ is the dye concentration at time *t*. Additional experimental observations are discussed in the Supporting Information document (see Heterogeneous Photocatalytic Degradation of Methylene Blue in the Supporting Information).

## Results and Discussion

### Material Characterization

#### Foam Morphology

**Figure 1:**
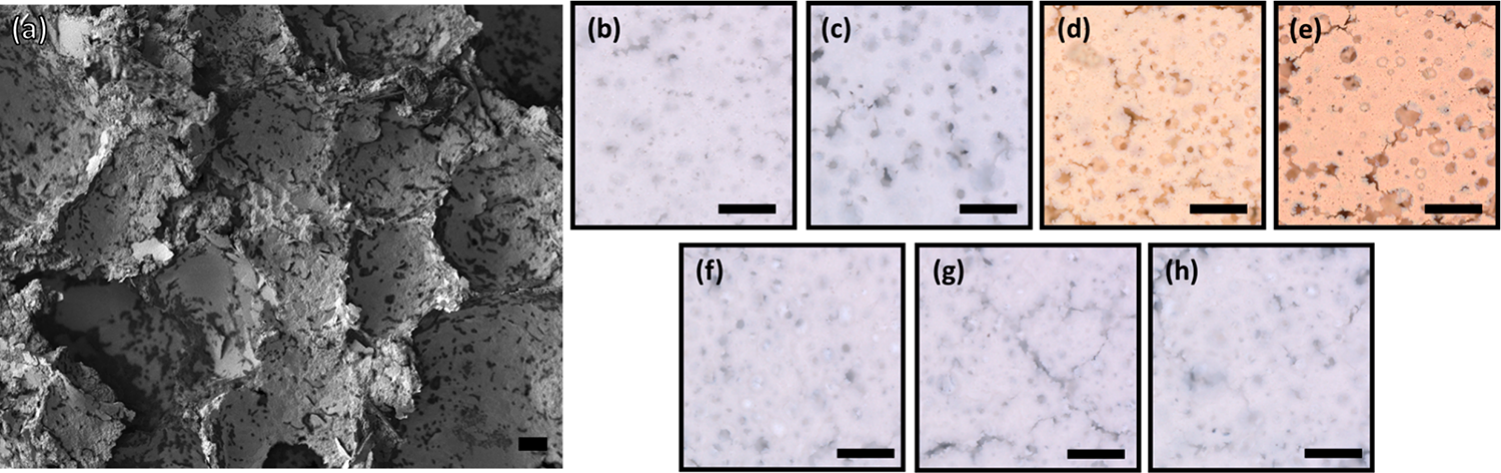
(a) SEM image of a cross-section of the foam depicting a closed-cell structure. Optical images of the titania foam film surface morphology at (b) room temperature and heated to (c) 100 °C, (d) 200 °C, (e) 300 °C, (f) 400 °C, (g) 500 °C, and (h) 600 °C. Scale bars are (a) 10 μm and (b–h) 200 μm.

Scanning electron microscopy (SEM) was used to inspect a cross-section of the foam, illustrating a macroporous closed-cell structure (Figure 1a). Macropores from the air bubbles were on the order of 10–60 μm in diameter. The foam formulation presented in this work differs from foams that Torres et al.^22^ fabricated in that no poly(acrylic acid) was used in the aqueous phase and glycerol was substituted for monoethanolamine in the oil phase as a surfactant. With these changes, a closed-cell macroporous foam structure was obtained with similar morphology to Torres’ closed-cell foams. Samples of the foam were doctor-bladed onto glass substrates and fired at various temperatures. Optical images of the foam surfaces show that a color change occurs at 200 and 300 °C as the organic material thermally decomposes, before turning white again at 400 °C (Figure 1b–f). As the temperature increases up to 500 and 600 °C, titania predominantly remains in the foam and no further color changes are observed (Figure 1g,h).

#### Viscosity

The foam was found to exhibit a shear thinning behavior. Measurements across a period of 192 h showed that apparent viscosity at shear rates below 0.5 s^–1^ did not change dramatically (range of ±35%) over that time (Figure S6). However, at higher shear rates, there was a significant increase in apparent viscosity at the 192 h mark. This could be attributed to phase separation from gravity-induced drainage over the duration of the experiment, causing the solid loading of the foam to increase with time.

#### TGA

**Figure 2:**
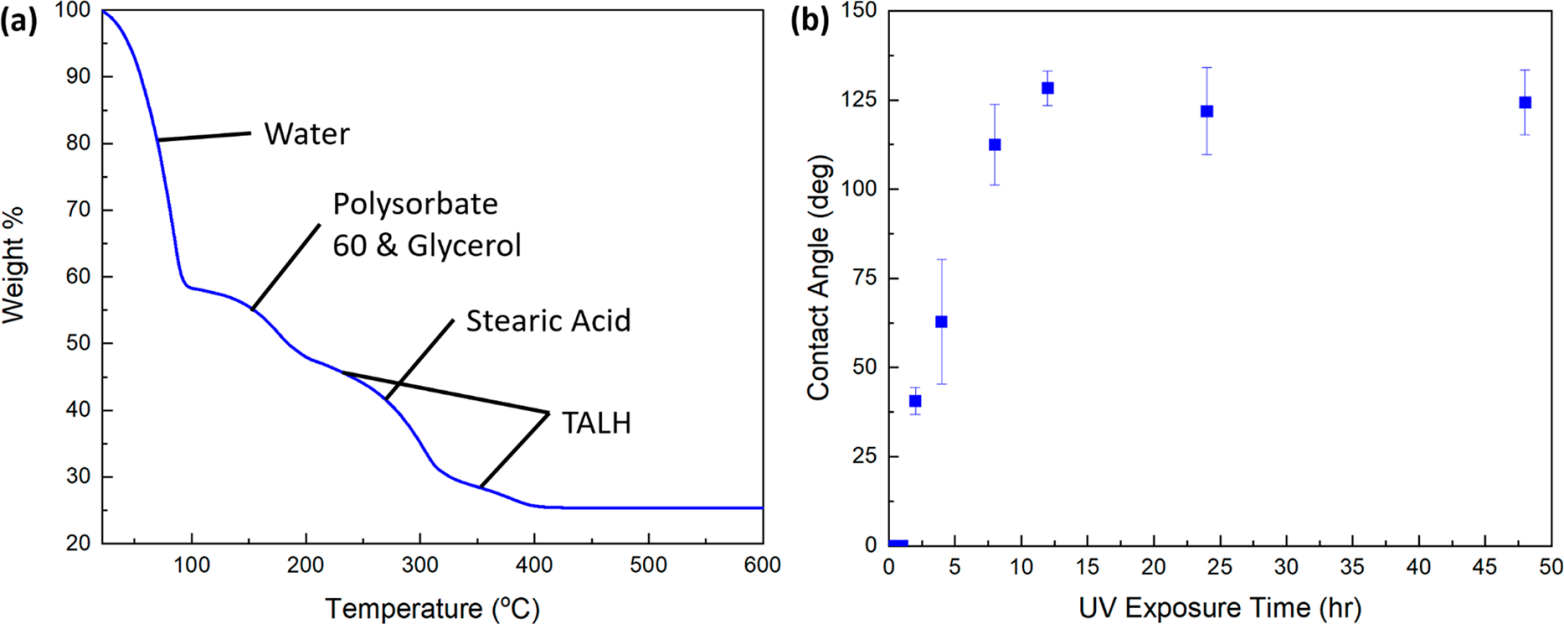
(a) Thermogravitmetric analysis curve of the titania foam. Organic decomposition points are labeled. (b) Contact angle of deionized water on titania foam as a function of ultraviolet (UV) exposure time for films of the TiO_2_ foam.

Thermogravimetric analysis (TGA) of the foam illustrates the change in mass due to the organic decomposition of the titania foam’s constituent materials during the heat-treating process (Figure 2a). The initial drop in weight corresponds to water evaporation. The curve at 150 °C indicates the P60 and glycerol decomposition. TALH decomposes and forms titania slowly over the temperature range from roughly 100 to 400 °C (Figure S3). At around 300 °C the stearic acid begins to decompose. At about 360 °C the TALH begins its final decomposition step and conversion to TiO_2_. The final weight at 500 °C is 25.3% of the original foam. Before sintering, the foam consists of a theoretical 20.6 wt % TiO_2_ particles and 12.6 wt % TALH. From the TGA curve of the TALH (Figure S7), approximately 14.4 wt % of the TALH solution is converted to TiO_2_ at 500 °C. This would lead us to assume that roughly 1.8 wt % of the final TiO_2_ particle loading would be directly from TALH conversion, for a final theoretical TiO_2_ wt % loading of 22.4 after heating to 500 °C. However, we observe that the experimentally measured final TiO_2_ loading is closer to 25.3 wt %. The difference between the theoretical and measured values could be explained by the initial TiO_2_ particles serving as nucleation sites on which the TALH molecules could grow new TiO_2_ at an increased rate compared to the TALH by itself, and that some organics did not completely decompose and burn off during the heating process.

#### Contact Angle

Films of the titania foam were doctor bladed onto glass slides at thicknesses of 300 μm and then fired in air at various temperatures from 100 °C up to 600 °C for 1 h. A titania film left in ambient conditions (23 °C) was used as a baseline comparison. Contact angle of DI water was measured on each of the films’ surface (Figure S8). Wetting of DI water droplets on the films’ surfaces was observed on each of the samples besides the one heated to 200 °C. The wetting on the room temperature and 100 °C samples is attributed to hydrogen bonding between the droplets and both the existing water in the foam and the polysorbate 60. A nearly superhydrophobic contact angle (147.9 ± 0.9°) was observed on the 200 °C sample. This can be attributed to the evaporation of the existing water in the foam and the decomposition of the polysorbate 60^33,34^ and the glycerol^35^ (Figure 2a), leaving a layer of hydrophobic stearic acid on the foam surface. This agrees with similar contact angle studies^36−38^ involving TiO_2_ films and stearic acid in which hydrophobicity was observed for samples heat-treated up to 250 °C. For samples fired to 300 °C and above, wherein the stearic acid thermally decomposes,^39^ the wetting was due to capillary action of the water in the hygroscopic titania foam matrix.

The effect of UV exposure on the foam with the contact angle of water was investigated. Doctor-bladed foam films were exposed to UV for up to 48 h. After 2 h of UV exposure time, the contact angle of DI water on doctor-bladed foam films began to increase from completely wetting to plateauing at a hydrophobic angle of approximately 124.8 ± 3.2° (Figure 2b). Previous studies have demonstrated that thin TiO_2_ films exhibit an increase in hydrophilicity upon UV exposure.^36,40−44^ We attribute the opposite trend in our observations to two factors: (1) the evaporation of the water in the foam over time and, decreasing the number of molecules available for hydrogen bonding, and (2) the UV radiation could oxidize and degrade the polysorbate 60 and glycerol,^45^ reducing their hydrophilicity and enabling the hydrophobic stearic acid to dominate surface force interactions, thus increasing the measured contact angle.

#### XRD

**Figure 3:**
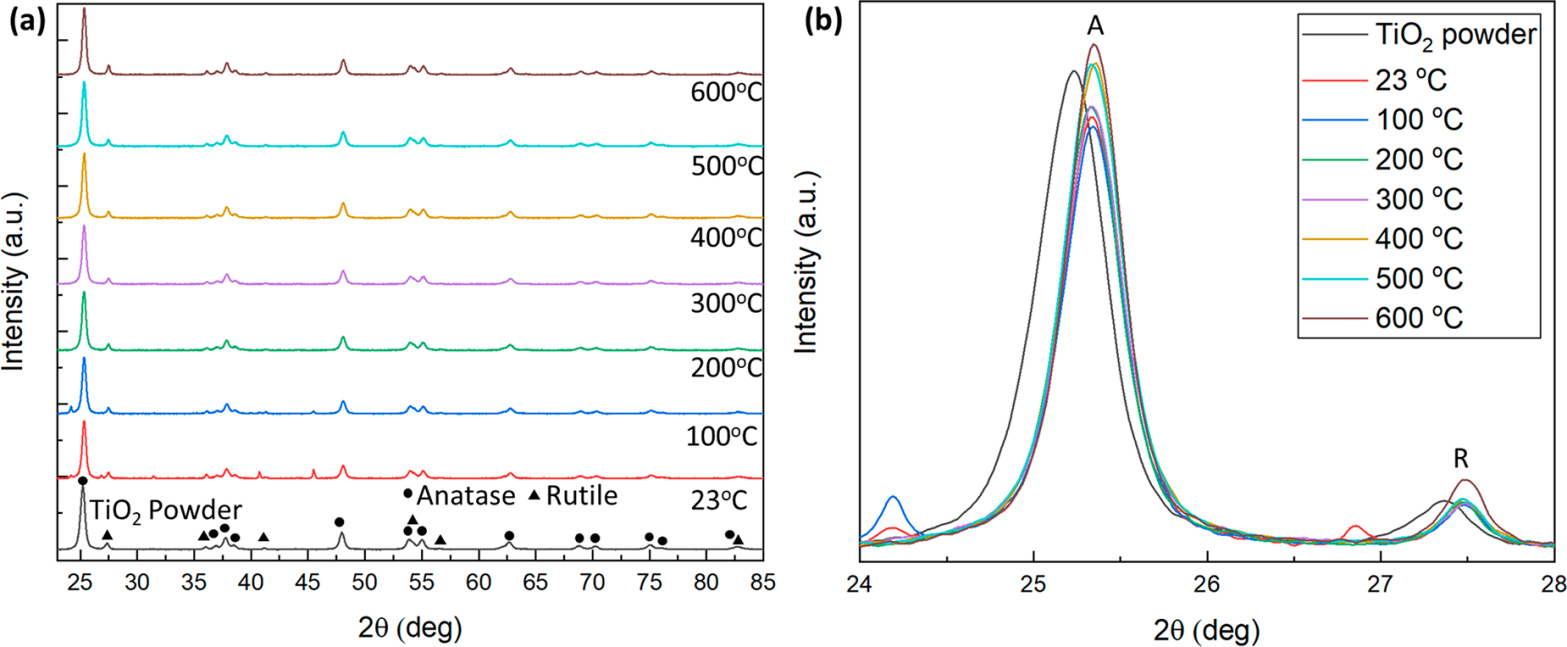
(a) X-ray diffraction patterns of the titania foam heat-treated at various temperatures up to 600 °C and the initial untreated titania particles. (b) Close-up of the main anatase (A) and rutile (R) diffraction peaks of the heat-treated foams.

X-ray diffraction was used to analyze how the crystal structure of the TiO_2_ in the foam changed as a function of heat-treament. The foam was doctor-bladed onto glass slides (thickness of 200 μm) and heated up to 600 °C for 1 h. Figure 3a shows the diffraction patterns for the heat-treated foam samples and the base titania particles. Plots of the % composition of anatase and rutile phases and % changes as functions of heat treatment are illustrated in the Supporting Information (Figure S9). The untreated primary titania particles were found to have an anatase:rutile phase ratio of 85%:15%. An increase of 3% and 1% in the amount of titania phase relative to the untreated primary titania particles is observed at 200 and 300 °C, respectively, before dropping to 0% at 400 °C. The opposite trend is observed for the rutile phase amount. The initial increase in the anatase phase is due to the thermal decomposition and conversion of TALH into TiO_2_.^46^ The relative increase in the rutile phase could be due to reversible phase changes of the small (∼2.8 nm), newly formed TiO_2_ crystallites.^47^ As the temperature increases to 500 °C a slight increase (1%) in anatase phase is observed, along with a corresponding drop in rutile phase. This can be attributed to the stabilization of the phases as the crystallite size increases. Finally, at 600 °C a 2% increase in the rutile phase is observed as the anatase begins to change to the more thermally stable rutile phase.^48,49^Figure 3b shows a close-up of the main diffraction peaks of the anatase and rutile phases of the titania. A shift to the right of the primary TiO_2_ particles is observed for both the anatase and rutile peaks. Additionally, two peaks are observed at 24.2° and 26.8° in the 23 °C sample and one sharper peak at 24.2° in the 100 °C sample. We speculate that these peaks are the result of metastable organic crystals forming during the decomposition of the polysorbate 60 and TALH.

#### UV–vis

**Figure 4:**
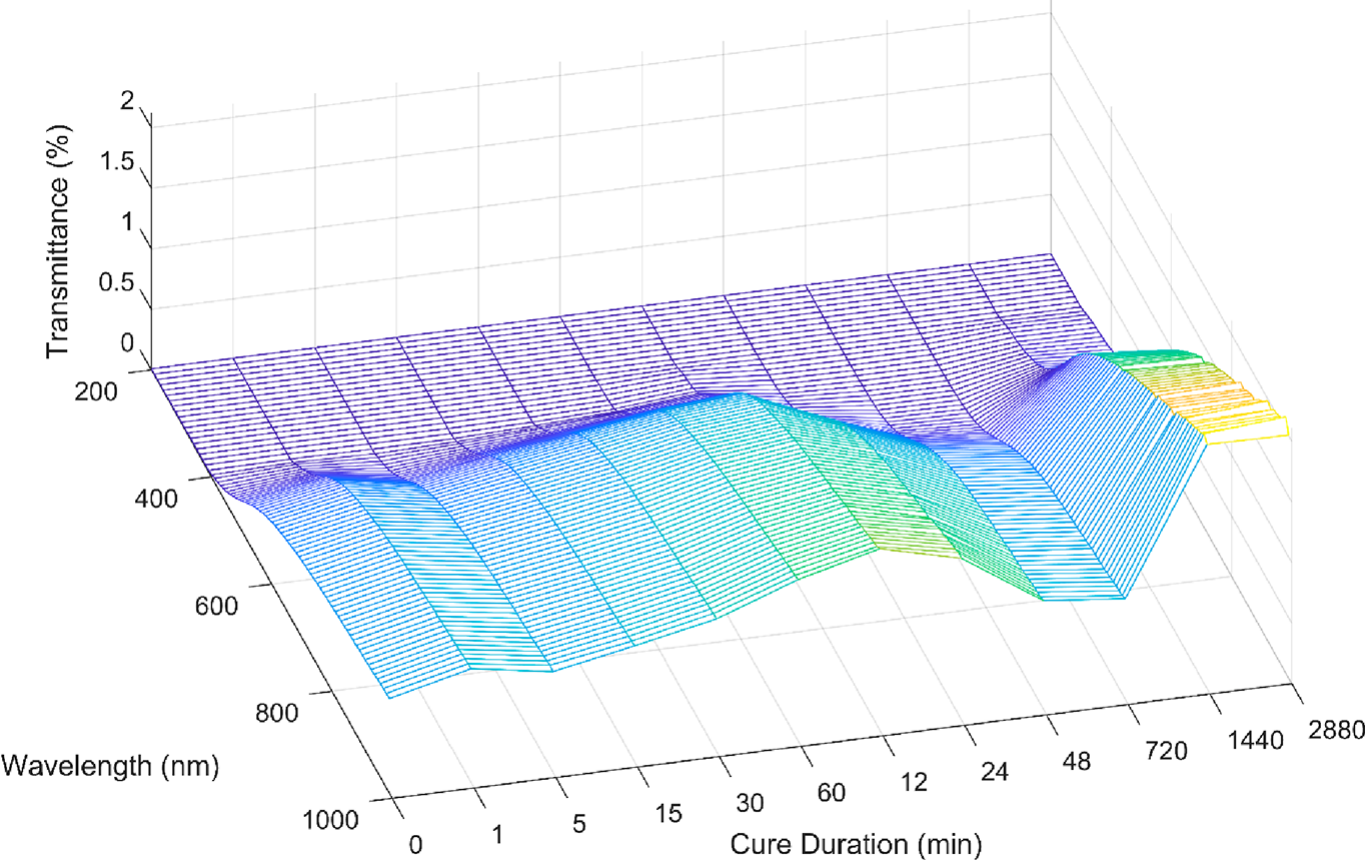
UV–vis transmittance spectra of the foam films after exposure to UV light for various times (Cure Duration).

Ultraviolet–visible light (UV–vis) spectroscopy was scanned across doctor-bladed foam films of uniform thickness (200 μm) that had been exposed to UV light for up to 48 h (2880 min, Figure 4). The films block almost all UV light (300–400 nm), achieving a transmittance of less than 0.2%. The visible light transmittance slowly increases with UV exposure time before decreasing at the 480 and 720 min marks. Then, an increase is observed again after 1440 min of UV exposure. A possible explanation for this downswing and upswing in transmittance could be due to two effects, respectively: (1) the TAHL in the foam reacts with UV light to degrade and form new titania crystals,^21,22,50^ increasing UV–vis absorption and (2) breakdown of the other organic molecules due to UV-induced oxidation over a longer time scale increases the amount of visible light capable of passing through the foam.

#### Coarsening

**Figure 5:**
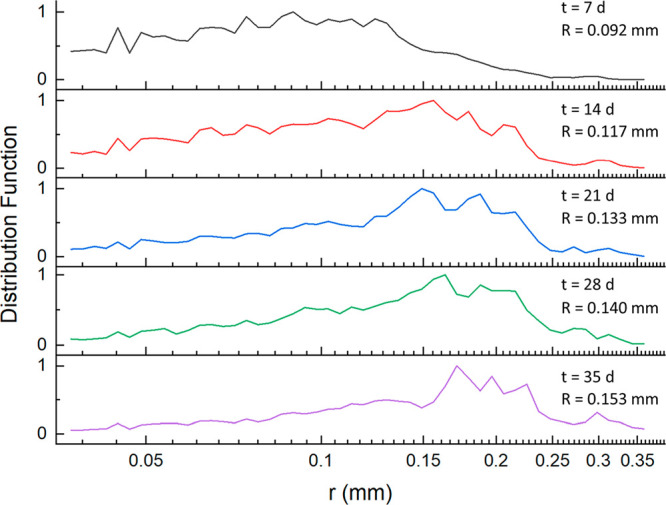
Histograms of bubble-size distributions (of radius *r*) for the titania foam over a period of 35 days. Inset shows the average bubble radius (*R*) and time (*t*).

Coarsening of the foam in Earth gravity over a period of 35 days illustrated how the foam’s average bubble radius increased over time (Figure 5). A unimodal distribution of the bubble radius occurred at early stages of the imaging (*t* = 7 d) before changing to a slight bimodal distribution (*t* > 7 d) with a small peak forming at the high end of the measured radii. The increase in average bubble radius is attributed to the molecular diffusion of air from smaller bubbles (relatively high pressure) to the larger bubbles (relatively low pressure) over time.

It is noted that the foam sample was weighed before and after the 35-day period and showed a 3.0% increase in mass. This could be explained by the hygroscopic nature of the constituent titanium dioxide.

### Printed Lines Analysis

**Figure 6:**
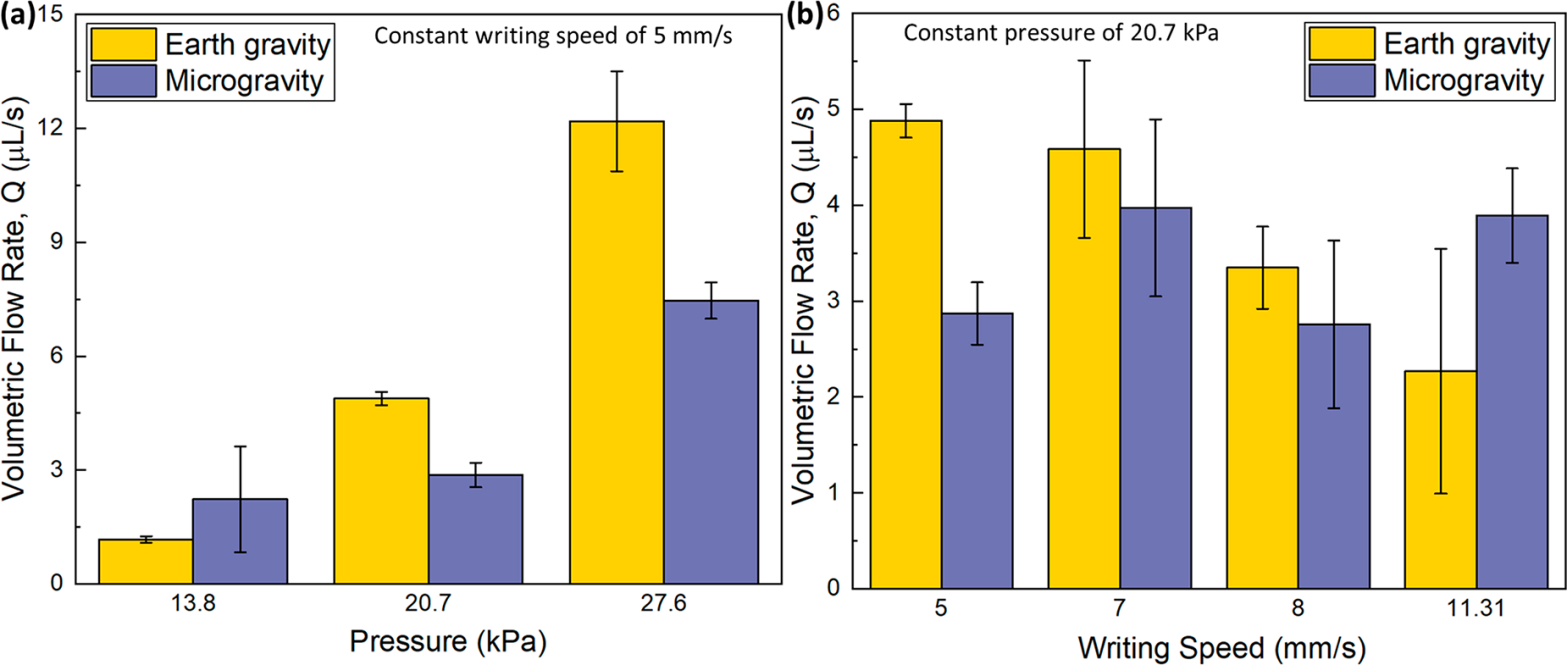
Volumetric flow rate of the printed foam as functions of (a) pressure (with a constant writing speed) and (b) writing speed (with a constant pressure) as printed in Earth gravity and in microgravity.

Volumetric flow rate, *Q*, of the foam during extrusion at various writing speeds and pressures was calculated and plotted for both Earth gravity and microgravity cases (Figure 6). Figure 6a shows *Q* as a function of extrusion pressure and a constant writing speed of 5 mm/s. *Q* increased as pressure increased for both gravity cases. Generally, *Q* was lower when printing in microgravity compared to printing in Earth gravity. At the lowest pressure, 13.8 kPa, Q was higher in microgravity than in Earth gravity. Figure 6b shows *Q* as a function of writing speed with a constant extrusion pressure of 20.7 kPa. In the Earth gravity cases, *Q* decreased as writing speed increased. However, in the microgravity cases *Q* was more varied, increasing as writing speed increased from 5 to 7 mm/s, then decreasing from 7 to 8 mm/s, before finally increasing from 8 to 11.31 mm/s, basically showing no trend versus writing speed (note the overlapping error bars).

**Figure 7:**
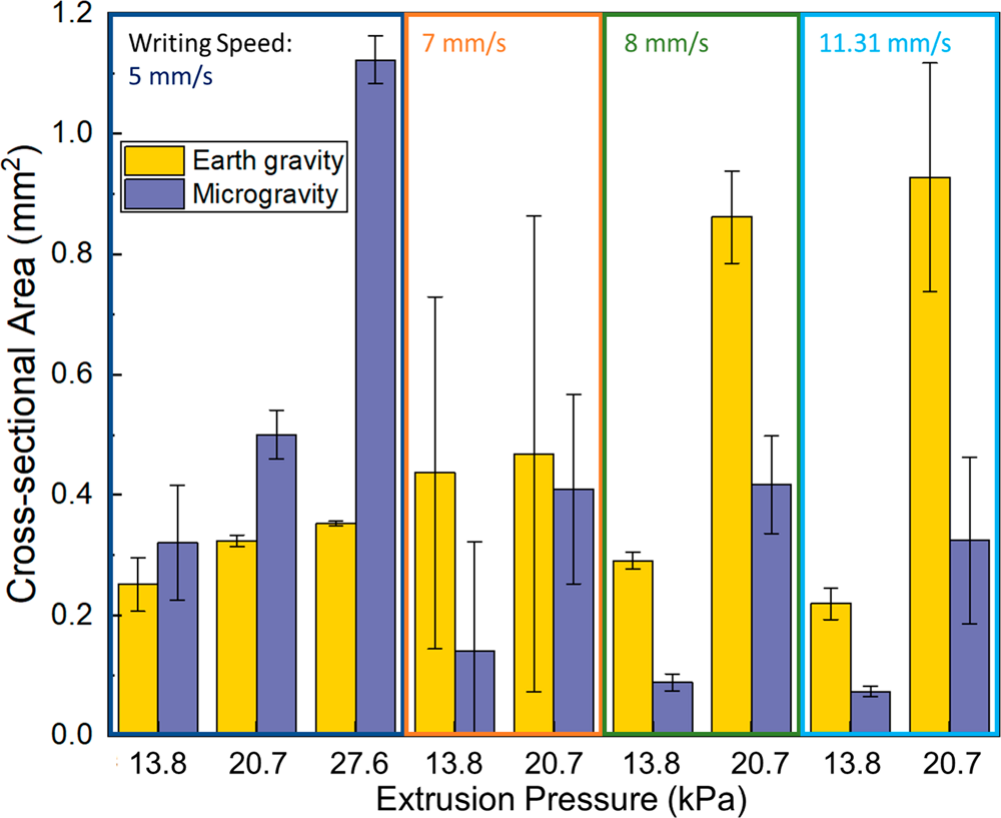
Average cross-sectional area of the printed lines as a function of extrusion pressure and writing speed for lines printed in Earth gravity and in microgravity.

Optical profilometry was used to characterize the physical dimensions of the printed lines of foam. Average cross-sectional area is reported for lines printed at pressures ranging from 13.8 to 27.6 kPa and writing speeds from 5 to 11.31 mm/s (Figure 7). At a writing speed of 5 mm/s, the average cross-sectional area is higher for lines printed in microgravity compared to lines printed in Earth gravity. However, at speeds of 7 mm/s and higher, the trend switches such that the cross-sectional area is higher in lines printed in Earth gravity compared to microgravity.

We observe that for the foam lines printed at 20.7 kPa in Earth gravity that as writing speed increases, the cross-sectional area increases. We suspect this is a result of the viscoelastic nature of the foam. As the foam extrudes and contacts the substrate, the surface forces acting between the foam and substrate act to “anchor” the underside of the deposited foam line to the substrate. At the same time, as the nozzle continues moving along the defined toolpath, stresses are induced in the foam as the foam is strained between the “anchored” point and the nozzle. At low writing speeds, these stresses are low and have a relatively large amount of time to relax. As writing speed increases, the foam is stretched more and thus the stresses acting on the foam are higher, and the relaxation time is shorter. These induced stresses in the longitudinal direction of the foam line can cause lateral deformation, leading to the resulting observed increase in cross-sectional area.

The average height of the profiles is depicted in the Supporting Information (Figure S10). The samples written at 7 mm/s exhibit a larger height value when printed in microgravity compared to Earth gravity. However, for all other writing speeds, the opposite is either illustrated or the error bars overlap significantly such that it is difficult to distinguish a trend. The same is true for the average width of the profiles (Figure S11). Samples printed at 7 mm/s show a larger width value when printed in microgravity compared to Earth gravity. The average value of the cross-sectional areas (Figure 7) is higher when printed in Earth gravity compared to microgravity, seemingly contradicting the data from Figures S10 and S11. However, we note that although the averages are higher for both extrusion pressures, the standard deviation bars overlap significantly, and thus more experimental testing is required to confirm these results for the samples printed at 7 mm/s.

We speculate that at writing speeds of 7 mm/s and above, the increased cross-sectional areas when printing in Earth gravity compared to microgravity are attributable to the addition of the gravitational force and increased spreading (line width) of the foam. However, at the slower writing speed of 5 mm/s, more surface interaction between the liquids in the foam and the substrate may allow for a better adherence to the glass substrate compared to the higher speeds. In microgravity (without the influence of a downward gravitational force or coarsening effects from gravity-induced drainage), this greater adhesive interaction may lead to increased extrusion of the foam from the nozzle as the surface force interactions aid in drawing the foam from the nozzle. Additionally, the lack of a gravitational force may allow the foam line to retain a more semicircular profile (larger cross-sectional area).^26^

Surface roughness was also analyzed (Figure S12). However, no obvious trends were observed in the data, suggesting that perhaps roughness of the printed foams is not dependent on writing parameters but instead on slight variations in the composition of the foam itself.

### Heterogeneous Photocatalytic Degradation of Methylene Blue

**Figure 8:**
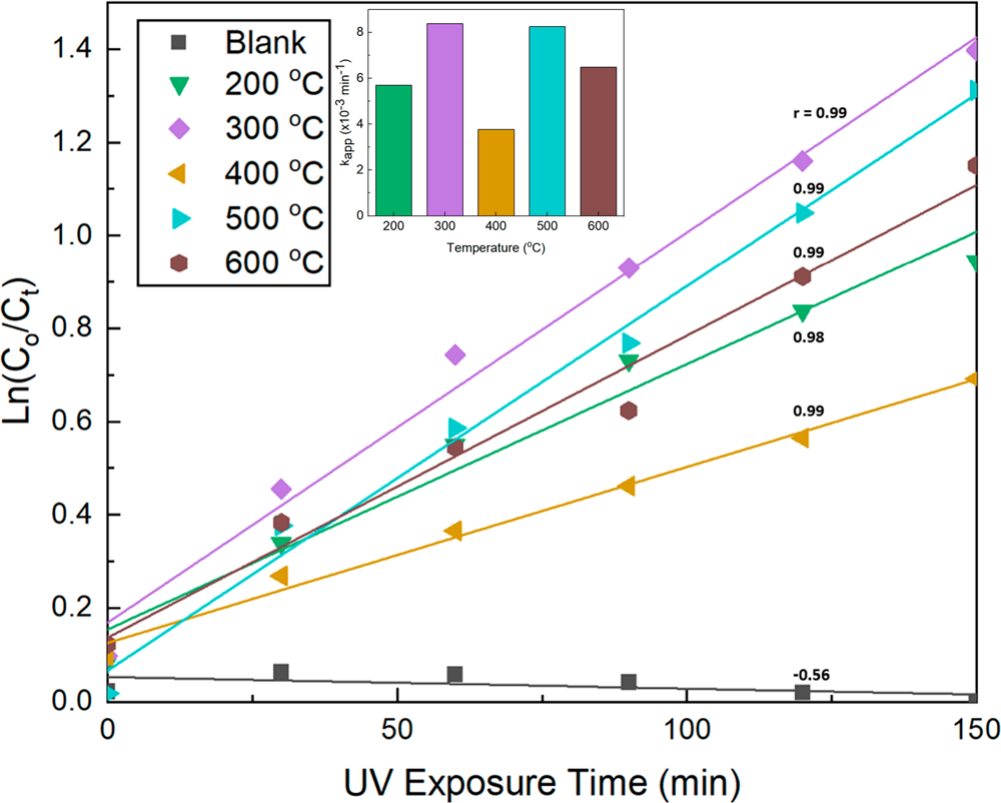
Linearized fits of the change in concentration of methylene blue as a function of time in the UV-exposed methylene blue (aq) solution with the foam films heat-treated at various temperatures. The slope of each fit is the apparent first-order degradation rate constant, *k*_app_. Pearson’s *r* is reported next to each linear fit. The inset plots the values of *k*_app_ for each temperature.

Heterogeneous photocatalytic degradation of methylene blue (aq) was carried out using doctor-bladed films (thickness of 200 μm) of the titania foam. The breakdown of an organic dye molecule such as methylene blue has been used as an indicator of the effectiveness of catalysts on the purification of water.^12,14,15,22,51−53^ The films were heat-treated at various temperatures and then submerged in the methylene blue solution (Figure 8). The submerged samples were exposed to ultraviolet light for up to 150 min. UV–vis absorbance measurements at 664 nm were used to monitor the change in concentration of methylene blue every 30 min. An optical image of the cuvettes illustrates the color changes observed after the UV exposure (Figure S13). The “blank” sample contained no TiO_2_ and was used to compare to the degradation rate of the foam samples. The inset in Figure 8 plots the apparent first-order degradation rate constants, *k*_app_, for each temperature. Table S1 lists the *k*_app_ values for each sample. In the “blank” sample, *k*_app_ has a slight negative linear trend, which is unexpected but could be due to slight experimental imperfections during the analysis. A slightly positive trend is usually expected in this scencario.^21,22,54^ For the foam samples, the *k*_app_ value increases with temperature until 400 °C, where there is a sharp decrease. We attribute this to two mechanisms: (1) the formation of new TiO_2_ crystallites on the surfaces of the existing titania primary particles due to the breakdown and formation of titania from the TALH molecules^21,22^ and (2) reversible phase changes of small TiO_2_ crystallites from the more photocatalysis-favorable anatase phase^14,15,55^ to the less active rutile phase as discussed in the XRD section. The small crystallites may adversely impact the photocatalytic degradation rate by disrupting the adsorption of the methylene blue molecules onto the primary titania particles. This corresponds to the TGA curve of the TALH (Figure S7), illustrating that the TiO_2_ formation completes after 400 °C, as well as the changes in detected amounts of anatase and rutile phases from XRD (Figure S9). At 500 °C, the rate constant increases, owing to the growth of the new titania crystals. Then, another decrease is observed at 600 °C. This is attributable to the tendency for titania phases to convert from anatase to rutile beginning around 600 °C.

## Conclusion

Titania foams have been successfully written in lines in microgravity and Earth gravity. Their resulting properties were compared, and it was found that the cross-sectional area of the printed lines is higher in microgravity than in Earth gravity at low writing speeds (5 mm/s). The opposite trend was observed as the writing speed increased. Samples had higher cross-sectional areas when printed in Earth gravity compared to microgravity. Additionally, various properties and behaviors of the foams were characterized. The shear-thinning foams displayed hydrophobic behavior when exposed to UV light and hydrophilic behavior when heated above 300 °C. Foam morphology and crystallinity were characterized with SEM and XRD, respectively. The foam was found to increase the amount of UV light blocked as a function of UV exposure time. Specifically, a 200 μm thick layer of foam blocked 99.8% of the UV radiation. Bubble coarsening was observed over a period of 35 days. Also, heterogeneous photocatalytic degradation of the methylene blue was used to demonstrate the potential of the foam as a water purification tool.

This work demonstrates the potential for an additively manufactured titania foam to be used in future space missions as a UV-shielding layer or water purification photocatalyst. Future studies should focus efforts on studying how performance changes for in-space additively manufactured devices (e.g., solar cells, water purifiers, UV shields) compared to those made on Earth. Understanding the effects of gravity, printing parameters (e.g., writing speeds and pressure), and postprocessing on the performance of titania foam films is an important step to developing efficient and sustainable manufacturing processes for long-term space flights where resource utilization management is of paramount importance.

## Supporting Information Available

Schematic of the printing system; profile scans of printed foam lines; description of the ANVOA analysis; Optical image of a foam line; apparent viscosity of the foam; thermogravimetric analysis plot of the titania precursor titanium bis (ammonium lactato) dihydroxide (TALH); contact angle of water on a layers of foam treated at various temperatures; percent composition of anatase and rutile phases of titania in the heat-treated foams; average profile height of the printed foam lines; average profile width of the printed foam lines; average profile roughness of the printed foam lines; discussion of some heterogeneous photocatalysis results; optical image of aqueous photocatalysis samples before and after UV treatment; table of apparent 1st-order degradation rate constants of the heat-treated foam samples during photocatalytic degradation of methylene blue (PDF)
