# Supplementary material for: Direct Writing of a Titania Foam in Microgravity for Photocatalytic Applications
Source: ACS Appl Mater Interfaces. 2023 Sep 28;15(40):47745–53. doi: 10.1021/acsami.3c09658 (PMC10571002; doi:10.1021/acsami.3c09658)
Supplement: Supplementary file 1 — am3c09658_si_001.pdf [file am3c09658_si_001.pdf]

Supporting Information

Direct Writing of a Titania Foam in Microgravity with Photocatalytic Applications

Authors

G. Jacob Cordonier,[1] Kyleigh Anderson,[1] Ronan Butts,[1] Ross O'Hara,[1] Renee Garneau,[1] Nathanael Wimer,[1] John M. Kuhlman,[1] Konstantinos A. Sierros[1*]

*Corresponding Author
Email: Kostas.Sierros@mail.wvu.edu

Author Affiliations

[1]Department of Mechanical and Aerospace Engineering, West Virginia University, Morgantown, West Virginia 26506, United States. *Email: kostas.sierros@mail.wvu.edu

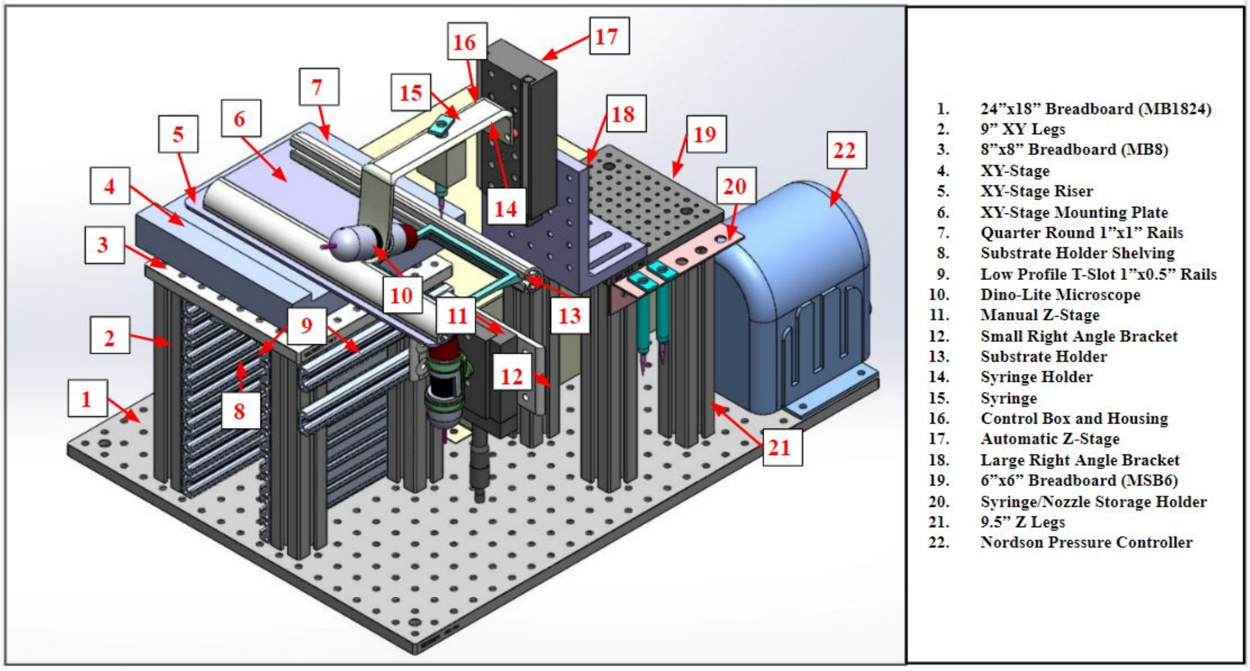

Figure S1. Schematic of the custom-built direct foam writing printer used to extrude the TiO_2 foam.

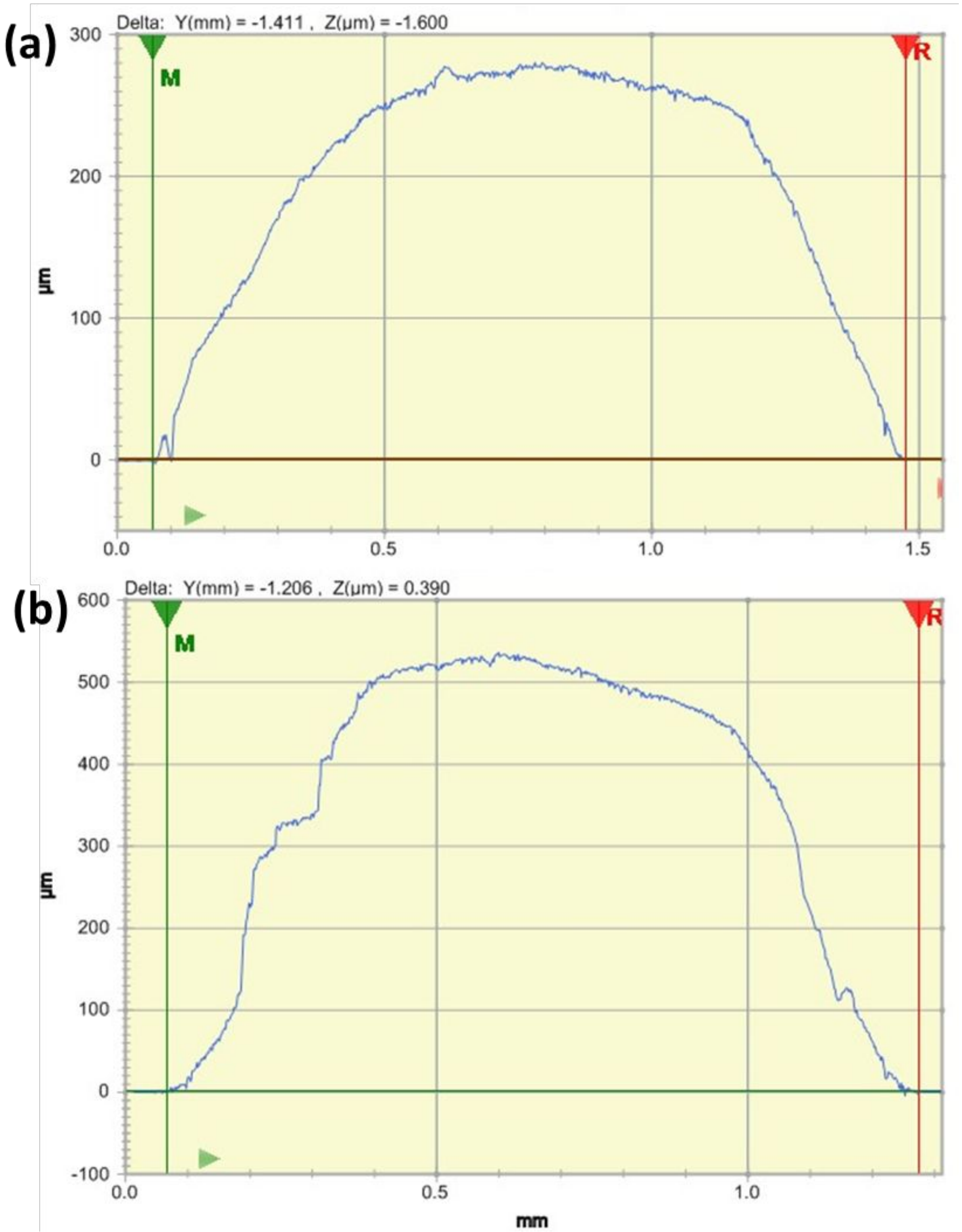

Figure S2 Example profiles of foam lines written in (a) Earth gravity at 11.31 mm/s and 13.8 kPa and (b) in microgravity at 8 mm/s and 20.7 kPa. Profiles were obtained using an optical profilometer.

Unbalanced ANOVA Analysis

Unbalanced Analysis of Variance (ANOVA) was performed on the data set because the data set in question had definite differences in total samples taken for each treatment (i.e., a unique set of levels of combined factors). Specifically, the hierarchical (also called “Type II”) sums of squares approach was utilized to assess the overall experimental results. Noting that 1-way ANOVA (i.e., ANOVA applied purely to single-factor influences on the system response) may be applied to any data set to help indicate the relative influence of each factor on a system’s response, a 1-way ANOVA was performed using MATLAB’s *anovan* function with the model set to ‘linear’ to analyze the system response in terms of the surface roughness of the Direct Foam Written sample against three factors: 1) the average extrusion pressure (“p avg”), 2) the extrusion nozzle’s translational speed relative to the substrate during printing (“write speed”), and 3) the local gravitational acceleration (“g local”). The results shown in Figure S3 indicate that all three factors have statistically significant influence on the resultant surface roughness of the test specimen. The results also indicate that of all the factors tested, it is likely that the extrusion pressure has the strongest impact on the surface roughness of the test specimens. It should be noted that the same 1-way ANOVA was applied to the same set of input factor data (i.e., extrusion pressure, write speed, and local gravitational acceleration) but tracked against different system responses (i.e., average line width, height, and cross-sectional area) with approximately the same results both in terms of predicted likelihood of error in rejection of the null hypotheses and in terms of priority of factors (i.e., extrusion pressure indicates the most influence and local gravitational acceleration indicates the least influence).

Analysis of Variance

Source	Sum Sq.	d.f.	Mean Sq.	F	Prob>F
p avg	210566.9	2	105283.5	60.62	1.35926e-24
write speed	45576.8	3	15192.3	8.75	1.11378e-05
g local	28961.8	1	28961.8	16.68	5.0695e-05
Error	988199.1	569	1736.7		
Total	1264477.8	575			

Figure S3. 1-way ANOVA applied to data set using MATLAB's anovan function with the model set to ‘linear.’

Based on the results of the 1-way ANOVA, a Type II hierarchical sum of squares unbalanced ANOVA was performed on the complete data set which assumed the hierarchy indicated by the 1-way ANOVA (i.e., evaluate extrusion pressure first, followed by write speed, followed by local gravitational acceleration). The hierarchical approach then allowed analysis of the subsequent three 2-way interactions which would then be followed by analysis of the single 3-way interaction. The results of the hierarchical sums of squares unbalanced ANOVA approach (shown in Figure S4) showed that all the system input factors and their resultant 2- and 3-way interactions had statistically significant influences on the system response (i.e., it would be extremely unlikely to be incorrect in asserting that each system factor and all possible permutations of interactions have some effect on the measured system response). The results also indicate that there is some significant interaction at all levels, particularly between the write speed and average extrusion pressure.

Analysis of Variance

Source	Sum Sq.	d.f.	Mean Sq.	F	Prob>F
p avg	210566.9	2	105283.5	125.41	1.11413e-45
write speed	45576.8	3	15192.3	18.1	3.23024e-11
g local	28961.8	1	28961.8	34.5	7.34846e-09
# p avg*write speed	302783.8	4	75696	90.17	4.7766e-59
p avg*g local	150492	2	75246	89.63	1.82021e-34
write speed*g local	23281	3	7760.3	9.24	5.64721e-06
# p avg*write speed*g local	45101.2	4	11275.3	13.43	1.86383e-10
Error	466770.5	556	839.5		
Total	1264477.8	575			

Figure S4. 3-way ANOVA applied to data set using MATLAB's anovan function with the model set to 'full' with a Type II sequential sum of squares approach

Ultimately, the results of the 1-way and hierarchical ANOVA tests support prioritizing control of the extrusion pressure first and translational speed of the nozzle head second in subsequent experiments. The results also support the idea that possibly greater resolution and/or a wider range of extrusion pressures should be prioritized when considering the basic economics of future experiments (e.g., if only 30 samples are possible during a single parabolic flight mission, then more discrete extrusion pressures and fewer discrete write speeds should be tested) to maximize experimental efficiency.

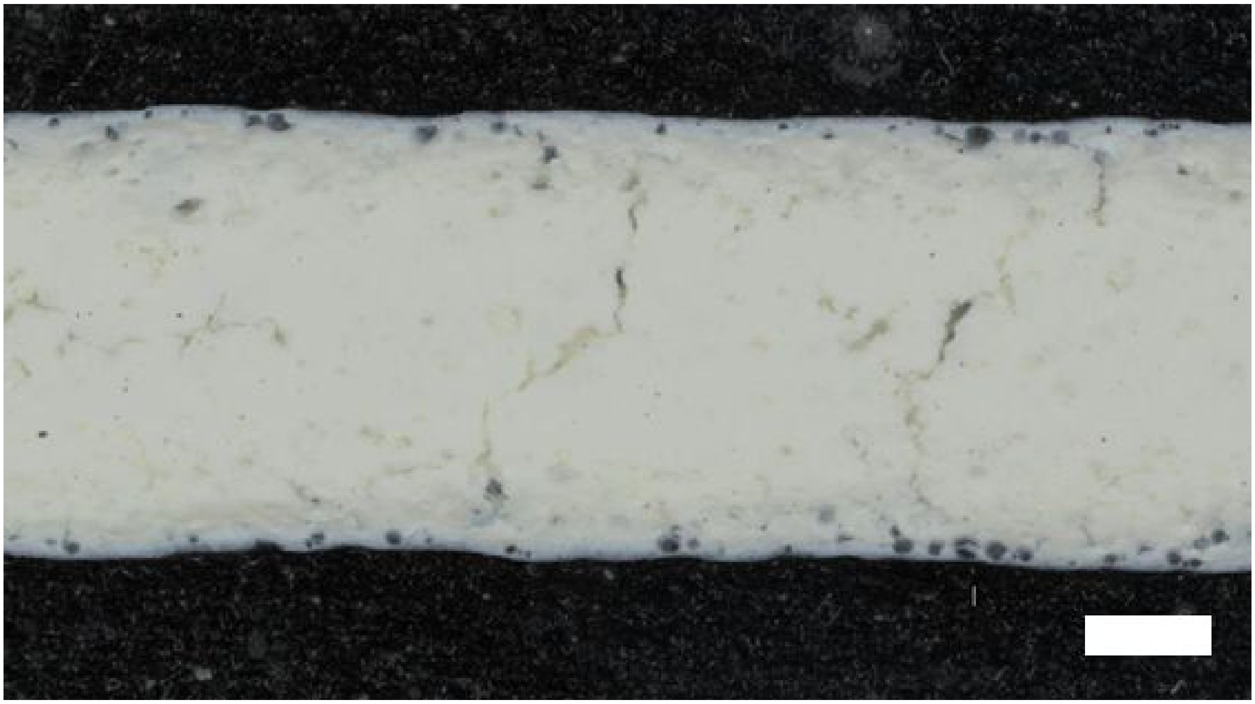

Figure S5. Optical image taken with a Keyence digital microscope of a foam line written in microgravity at 5 mm/s and 27.6 kPa. Scale bar is 500 µm.

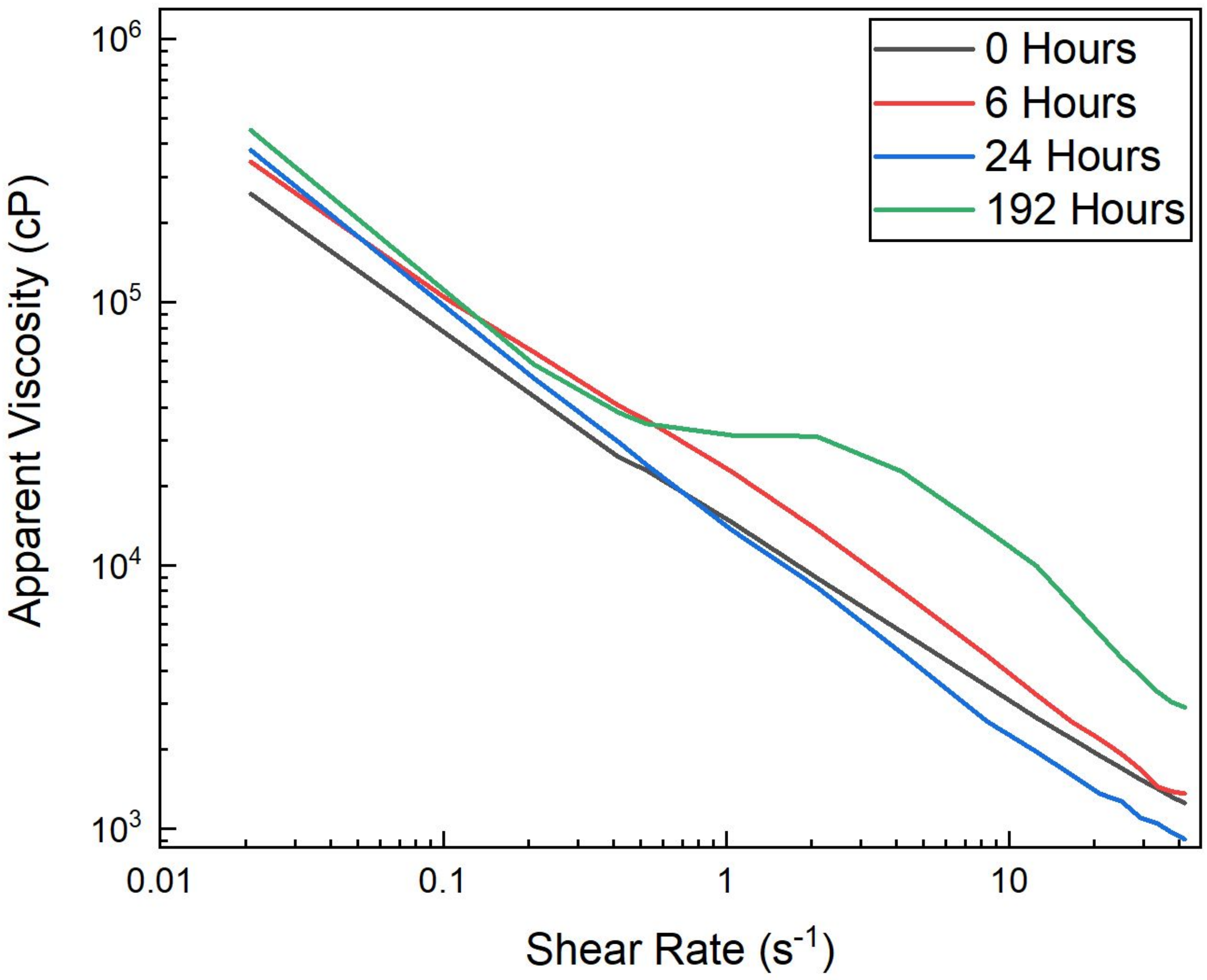

Figure S6. Apparent viscosity of the foam as a function of shear rate at various ageing times.

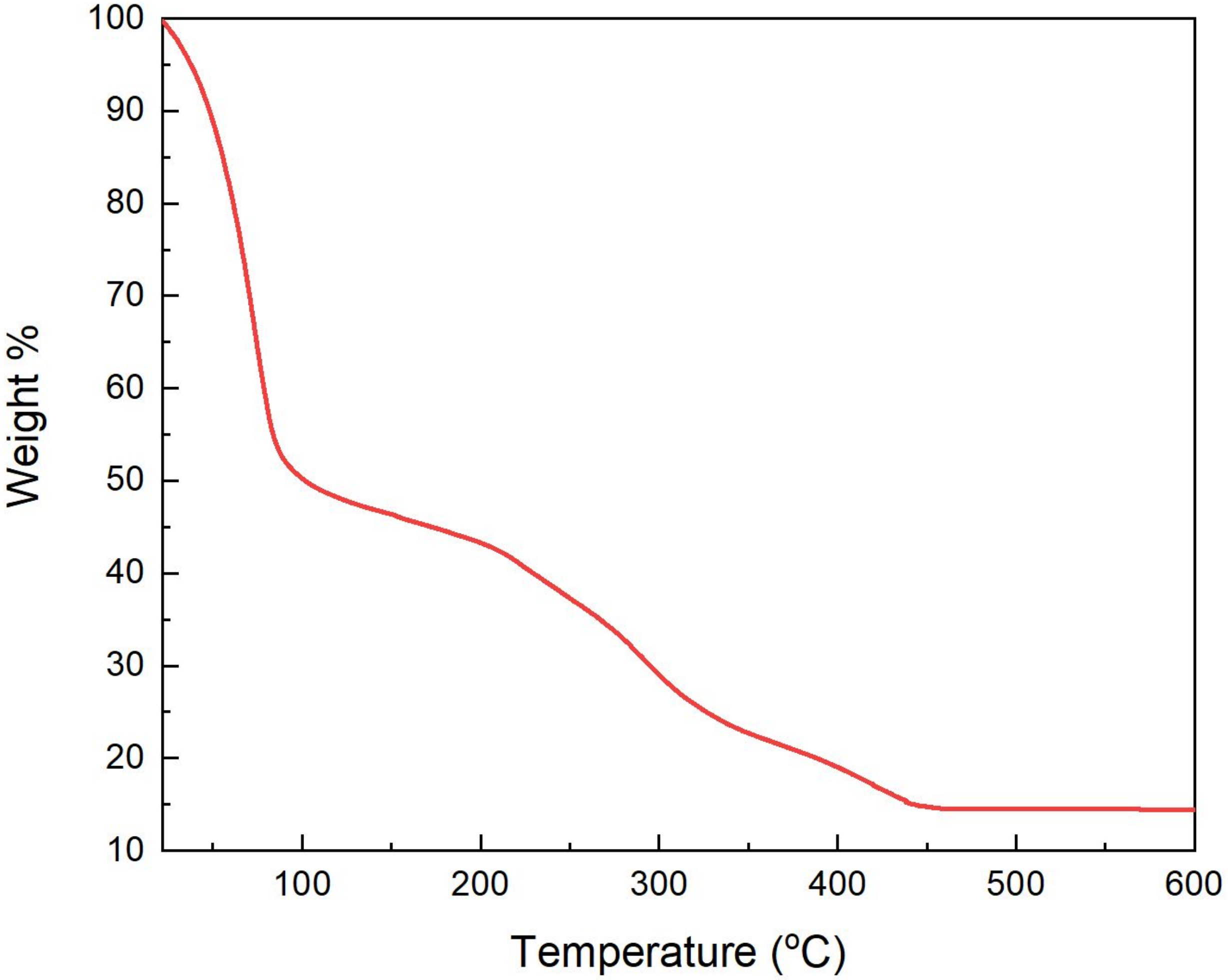

Figure S7. Thermogravimetric curve of the titania precursor titanium bis (ammonium lactato) dihydroxide (TALH) with a heating rate of 2 °C/min. The large initial drop in weight is the water evaporation, followed by organic decomposition and conversion to titania. The final weight percent remaining is 14.4%.

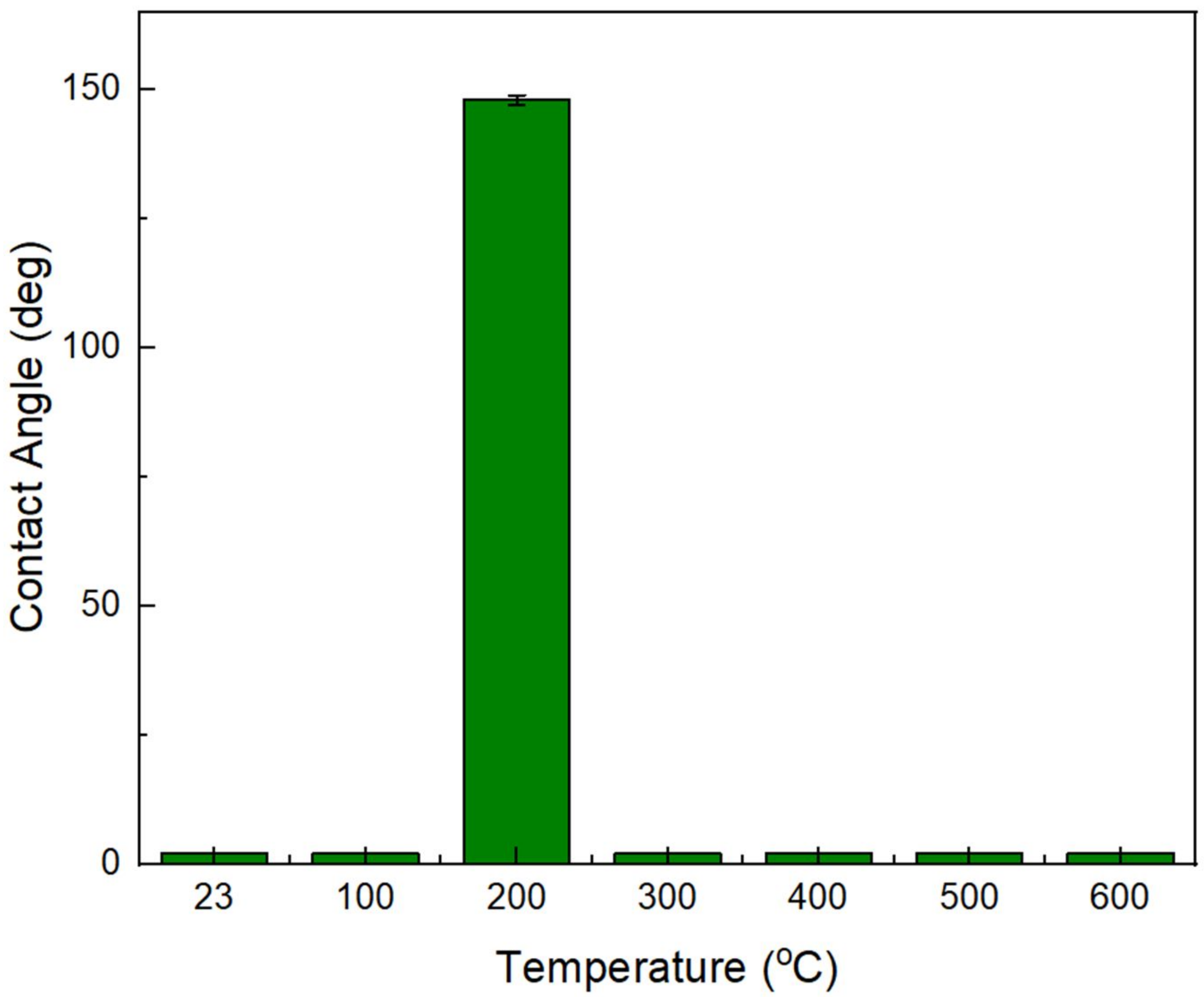

Figure S8. Contact angle of deionized water on doctor-bladed films of the foam as a function of firing temperature. Samples were fired for 1 hour.

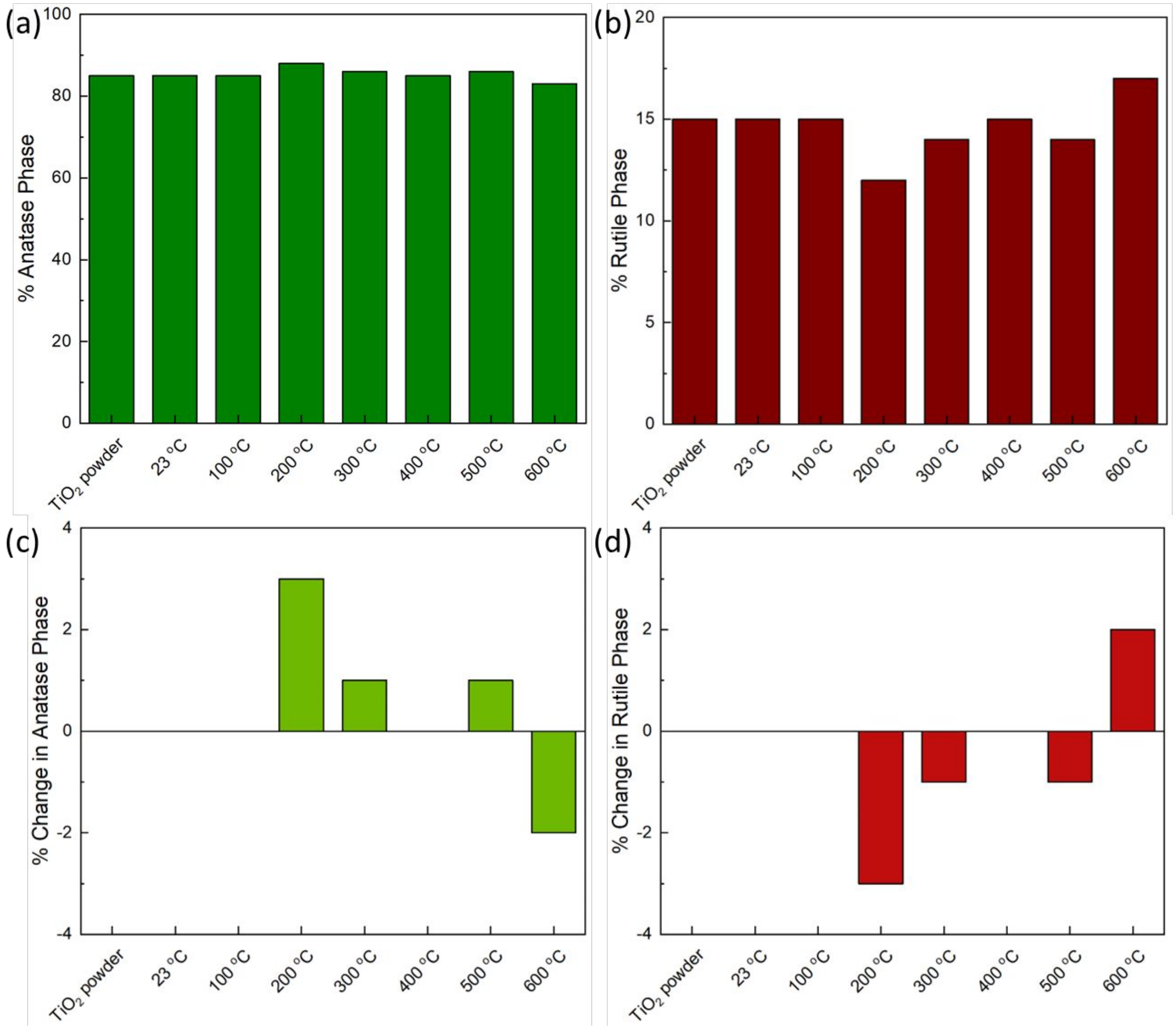

Figure S9. % Composition of (a) anatase and (b) rutile phases in the heat-treated titania foam film samples. % Change in (a) anatase and (b) rutile phases compared to the untreated primary titania particles.

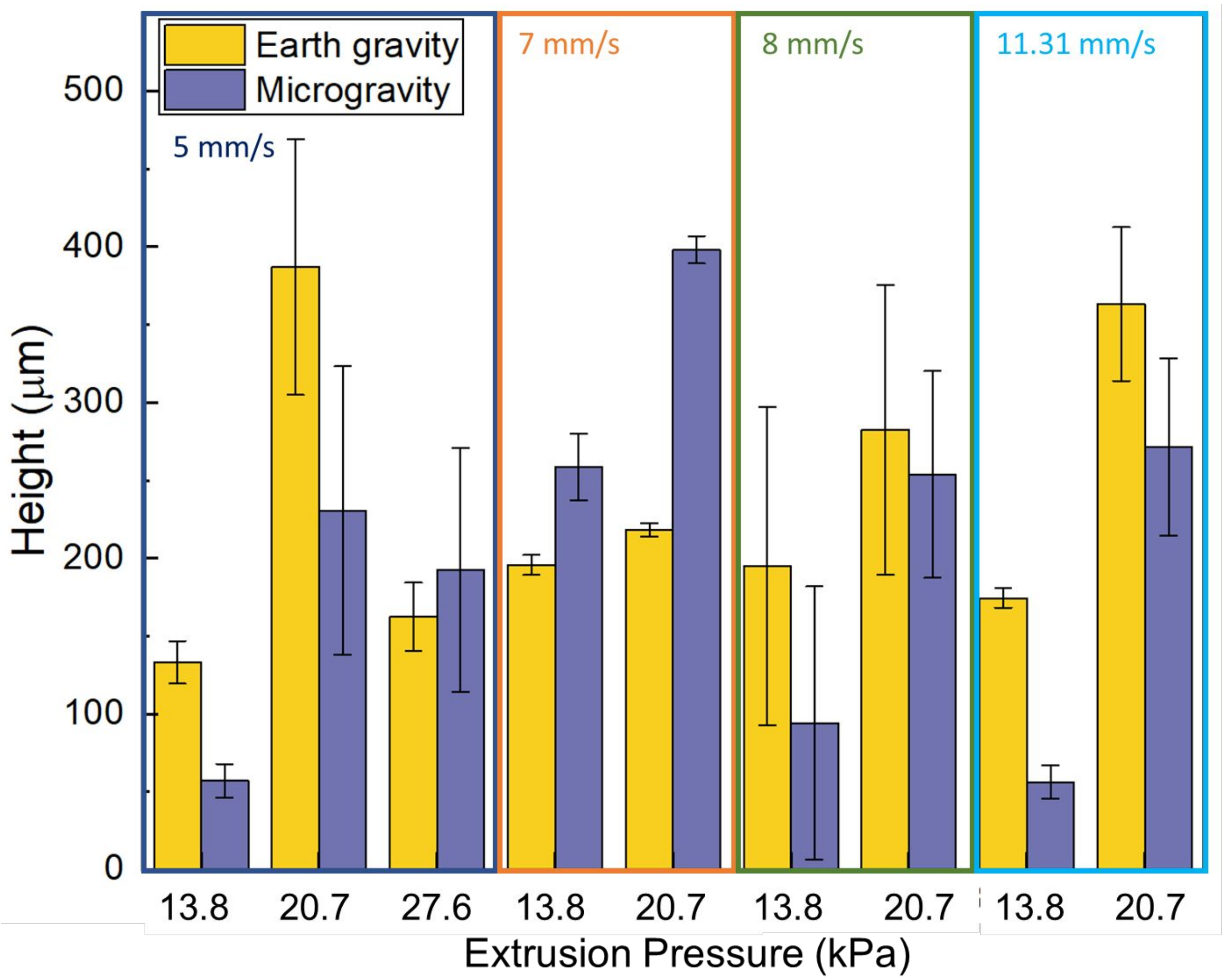

Figure S10. Average profile height of the titania foam lines printed in Earth gravity and microgravity as functions of extrusion pressure and writing speed.

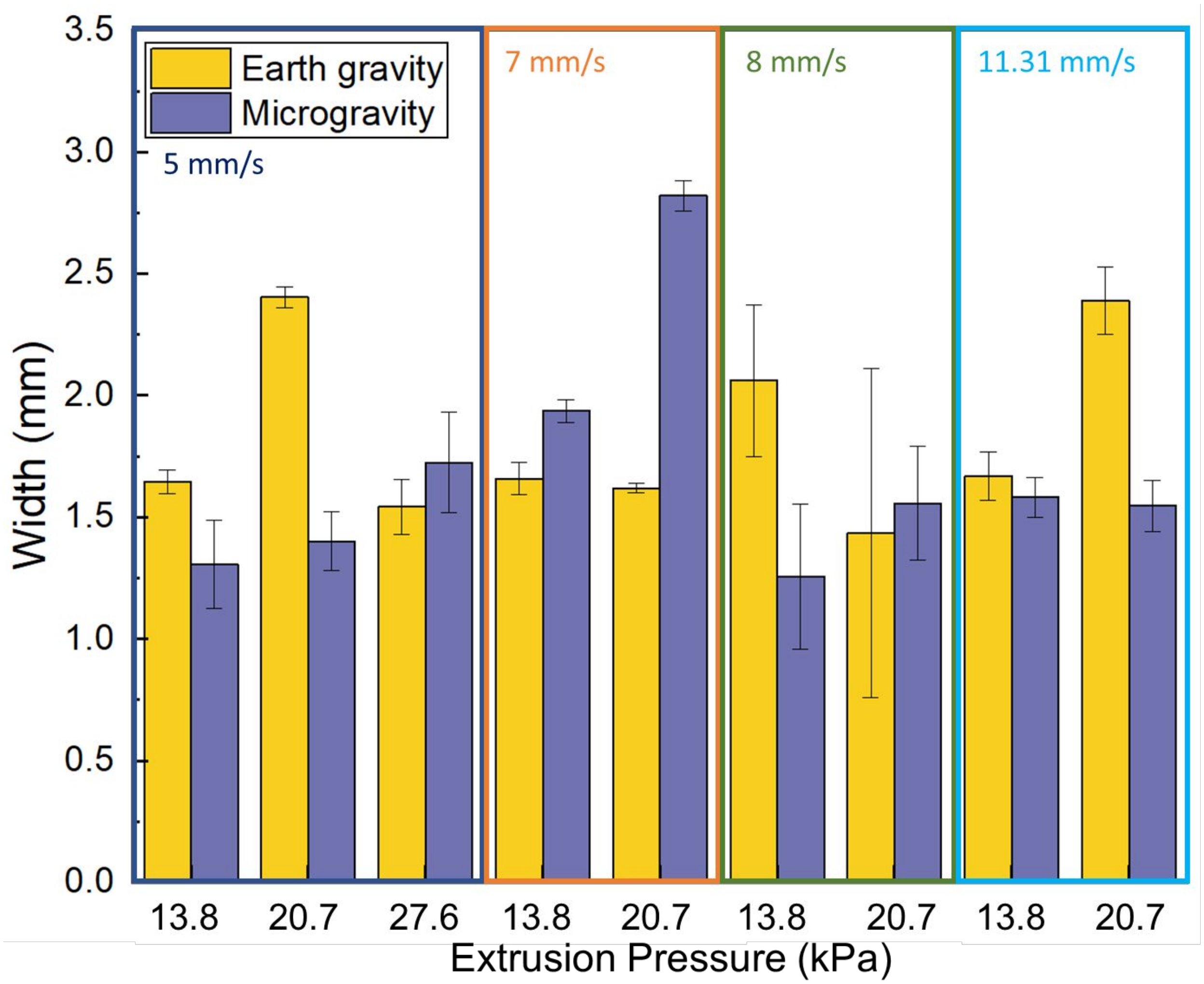

Figure S11. Average profile width of the titania foam lines printed in Earth gravity and microgravity as functions of extrusion pressure and writing speed.

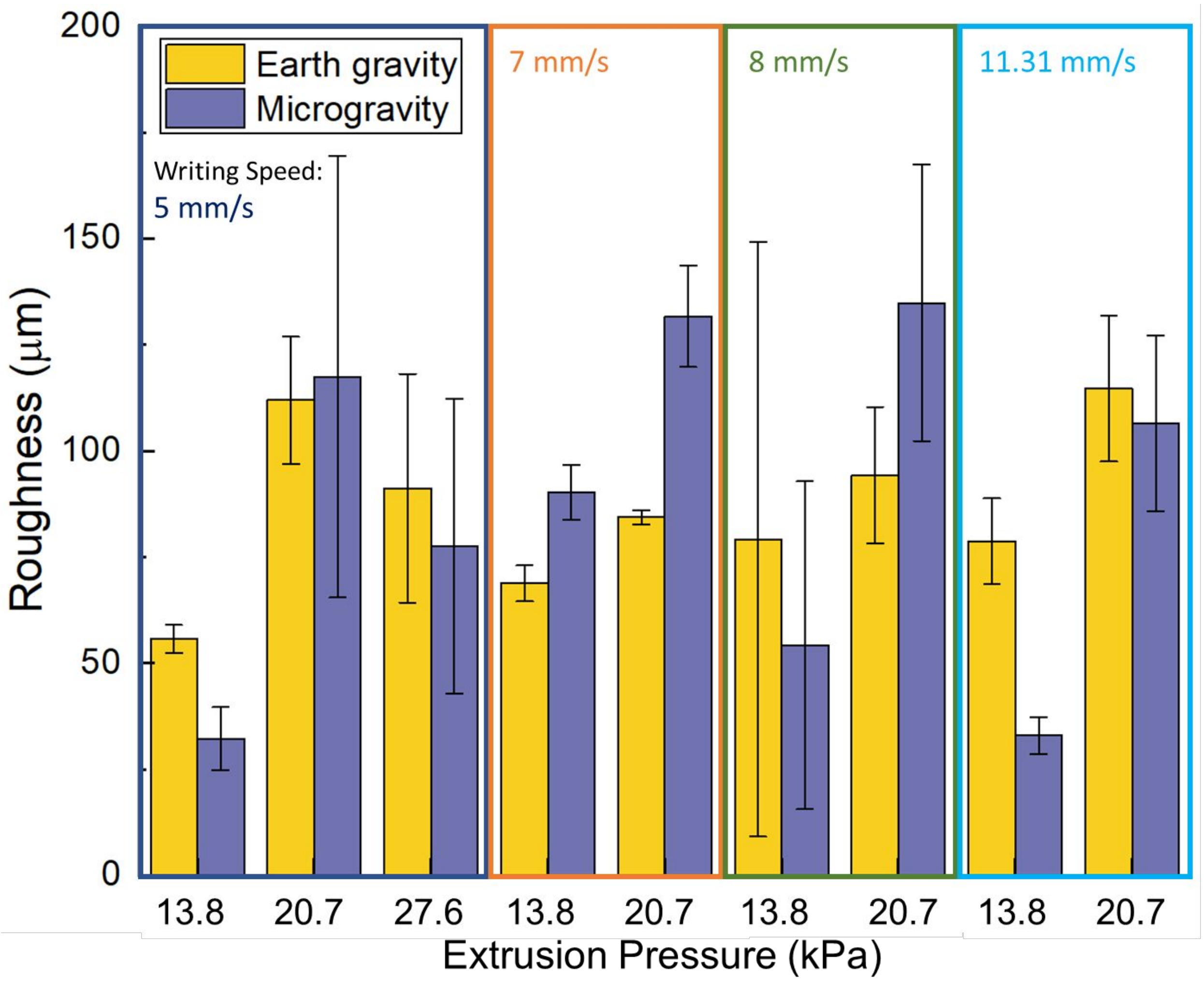

Figure S12. Average profile roughness of the titania foam lines printed in Earth gravity and microgravity as functions of extrusion pressure and writing speed.

Heterogeneous Photocatalytic Degradation of Methylene Blue

The authors note that during this experiment, samples of the doctor-bladed foam left at room temperature and heat treated to 100 °C for one hour each were examined. However, both samples delaminated from their respective glass slides and partially dissolved in the methylene blue solutions, since they had not been sufficiently cured at these temperatures, forming suspensions (Figure S10). Since suspensions violate the Beer-Lambert assumptions, the absorbance results were omitted from the results.

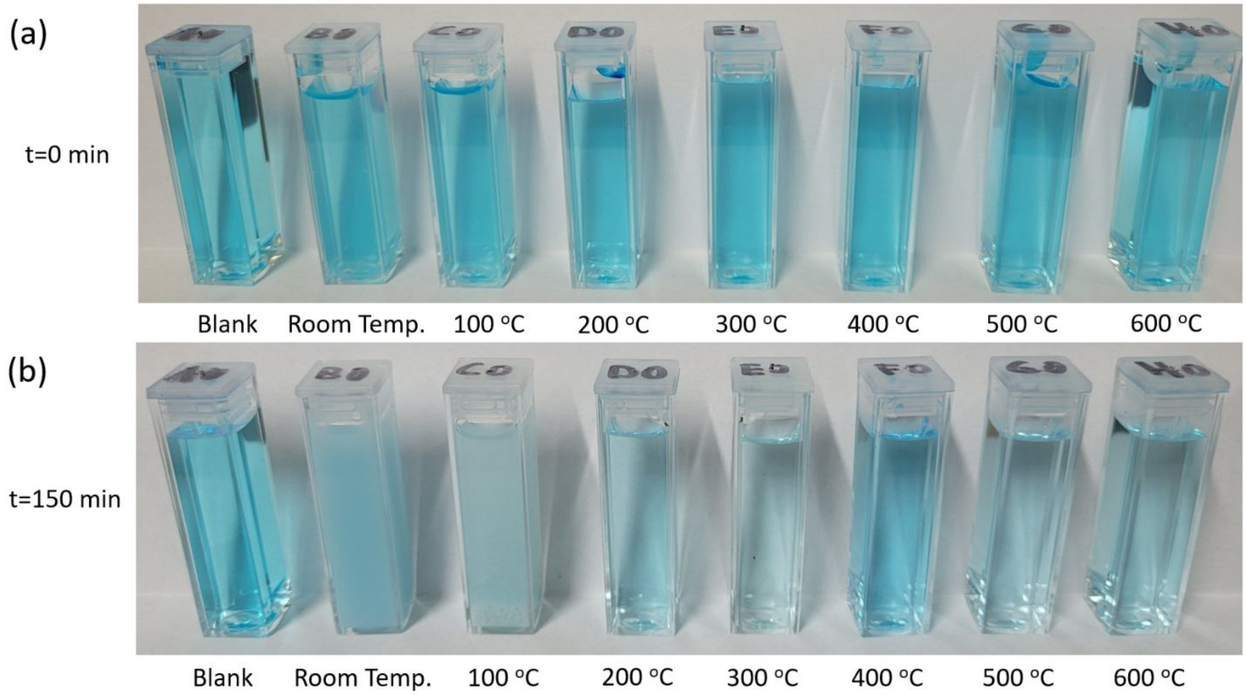

Figure S13. Optical images of samples of methylene blue (aq) before (a) and after (b) ultraviolet light exposure for 150 min with the titania foams.

Table S1. Apparent First-Order Degradation Rate Constant, k_{app}.

Sample	k_{app} (x10^{-3} min^{-1})
**Blank**	-0.25
**Room Temp.**	--
**100 °C**	--
**200 °C**	5.69
**300 °C**	8.38
**400 °C**	3.77
**500 °C**	8.25
**600 °C**	6.48
